# Supplementary material for: Resveratrol Analogues as Dual Inhibitors of Monoamine Oxidase B and Carbonic Anhydrase VII: A New Multi-Target Combination for Neurodegenerative Diseases?
Source: Molecules. 2022 Nov 13;27(22):7816. doi: 10.3390/molecules27227816 (PMC9694798; doi:10.3390/molecules27227816)
Supplement: Supplementary file 1 [file molecules-27-07816-s001.zip › molecules-1978837-supplementary.pdf]

molecules

Supplementary Materials

Resveratrol Analogues as Dual Inhibitors of Monoamine Oxidase B and Carbonic Anhydrase VII: A New Multi-Target Combination for Neurodegenerative Diseases?

Simone Carradori [1], Marialuigia Fantacuzzi [1], Alessandra Ammazzalorso [1], Andrea Angeli [2], Barbara De Filippis [1,*], Salvatore Galati [3], Anél Petzer [4], Jacobus P. Petzer [4], Giulio Poli [3], Tiziano Tuccinardi [3], Mariangela Agamennone [1] and Claudiu T. Supuran [2]

[1] Department of Pharmacy, University "G. d'Annunzio" University of Chieti-Pescara, via dei Vestini 31, 66100 Chieti, Italy
[2] Neurofarba Department, University of Florence, 50019 Sesto Fiorentino, Italy
[3] Department of Pharmacy, University of Pisa, Via Bonanno 6, 56126 Pisa, Italy
[4] Pharmaceutical Chemistry and Centre of Excellence for Pharmaceutical Sciences, North-West University, Potchefstroom 2520, South Africa
* Correspondence: barbara.defilippis@unich.it; Tel.: +39-087-1355-4685

Figure S1. The catalytic site of MAO-B in complex with safinamide.
Figure S2. The catalytic site of MAO-B in complex with and RSV.
Figure S3. The catalytic site of MAO-A in complex with harmine.
Figure S4. The catalytic site of MAO-A in complex with RSV.
Figure S5. Minimized average structure of hCA VII (PBD code: 3MDZ) in complex with compound 4 superimposed to hCA I X-ray structure (PDB code: 1AZM).
Figure S6. Minimized average structure of hCA VII (PBD code: 3MDZ) in complex with compound **4** superimposed to hCA VA homology model.
Figure S7. Minimized average structure of hCA VII (PBD code: 3MDZ) in complex with compound **4** superimposed to hCA VB homology model.
Table S1. Linear Interaction Energy results for the four analysed ligand-protein complexes of compound **4**.

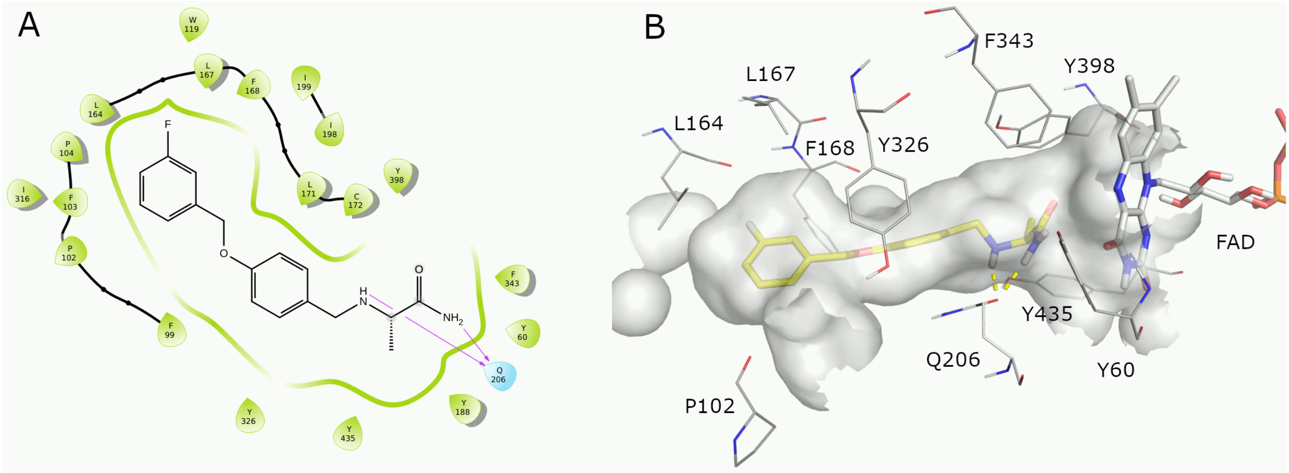

Figure S1. The catalytic site of MAO-B in complex with safinamide. (A) 2D ligand interaction diagram; (B) best docked pose of safinamide (yellow sticks) with MAO-B surrounding residues (grey lines). The crystallographic ligand of MAO-B, safinamide, mediates hydrophobic interactions in the hydrophobic cavity, in particular with F103, L164, L167, L171, I199, F343, Y398 and FAD, and 2 H-bonds with the side chain of Gln206. The docking score of safinamide is -10.375 kcal/mol.

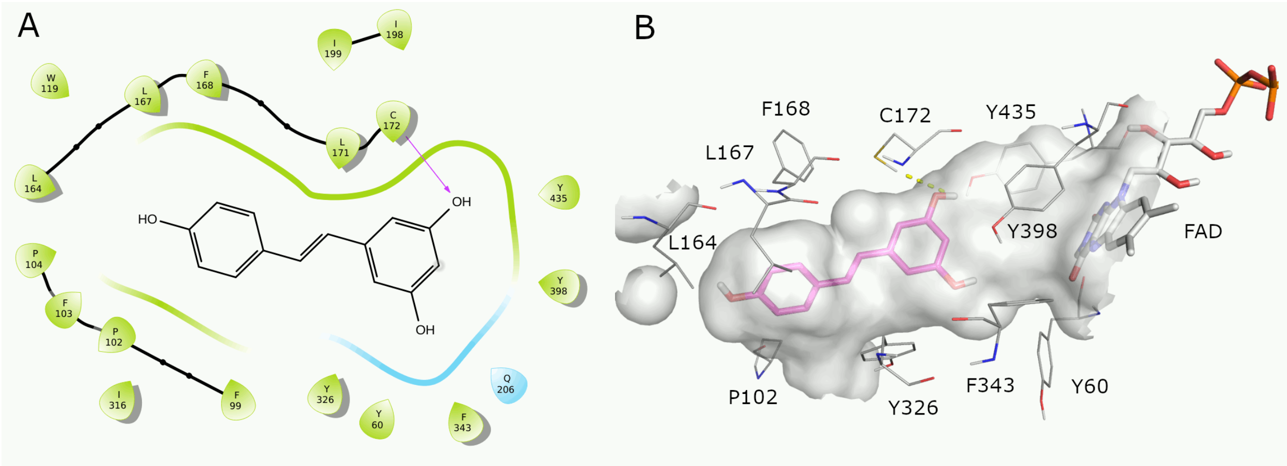

Figure S2. The catalytic site of MAO-B in complex with RSV. (A) 2D ligand interaction diagram; (B) best docked pose of RSV (magenta sticks) with MAO-B surrounding residues (grey lines). The analysis of the docked poses of RSV shows that the ligand stilbene scaffold is located in the hydrophobic region of MAO-B. The hydroxyl in 5 position makes an H-bond with C172. At the same time, the OH groups in 3 and 4′ occupy the hydrophobic region determining an unfavourable contribution to the docking score and probably, explaining the limited activity of this compound as MAO inhibitor.

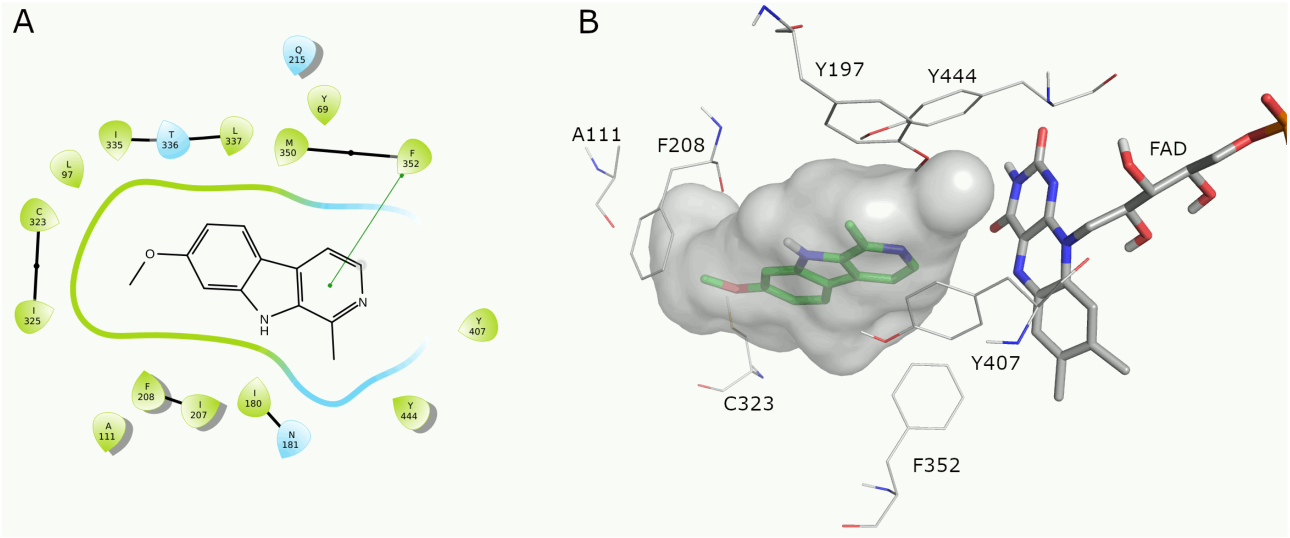

Figure S3. The catalytic site of MAO-A in complex with harmine. (A)2D ligand interaction diagram; (B) best docked pose of harmine (green sticks) with MAO-A surrounding residues (grey lines). Harmine, the crystallographic ligand of MAO-A, occupies the hydrophobic region and makes π- π interaction with Phe352 (**Figure S2 A,B**). The analysis of RSV in MAO-A revealed that the three hydroxyls are external to the hydrophobic region, and there is a π-π interaction with Phe208, that contributes to better positioning of RSV in MAO-A with respect to MAO-B, in accordance with the biological data (**Figure S2 C,D**).

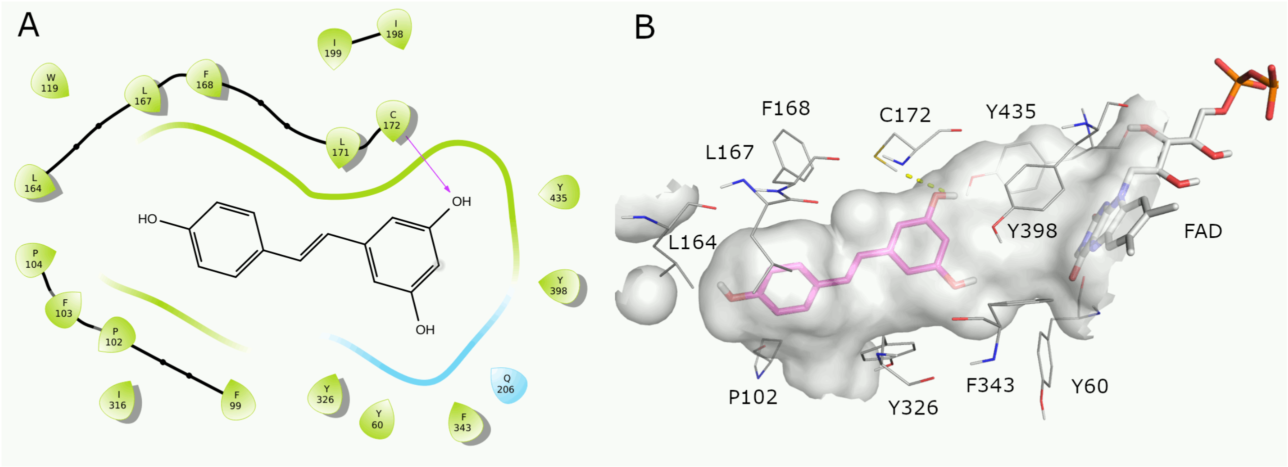

Figure S4. The catalytic site of MAO-A in complex with RSV. (A) 2D ligand interaction diagram; (B) best docked pose of RSV (magenta sticks) with MAO-A surrounding residues (grey lines). The analysis of RSV in MAO-A revealed that the three hydroxyls are external to the hydrophobic region, and there is a π- π interaction with F208, that contributes to better positioning of RSV in MAO-A with respect to MAO-B, in accordance with the biological data.

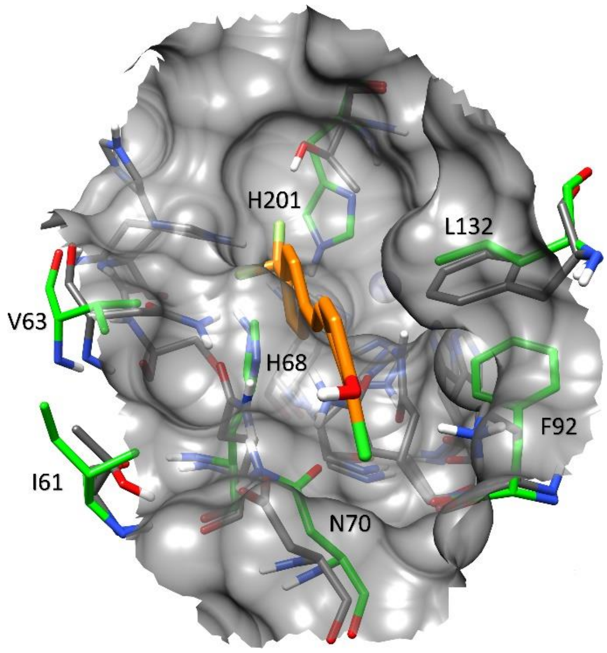

Figure S5. Minimized average structure of hCA VII (PBD code: 3MDZ) in complex with compound **4** superimposed to hCA I X-ray structure (PDB code 1AZM). hCA I non-conserved residues are labelled and shown in green. The other residues of hCA I and hCA VII are shown in grey. The protein surface is shown in dark grey.

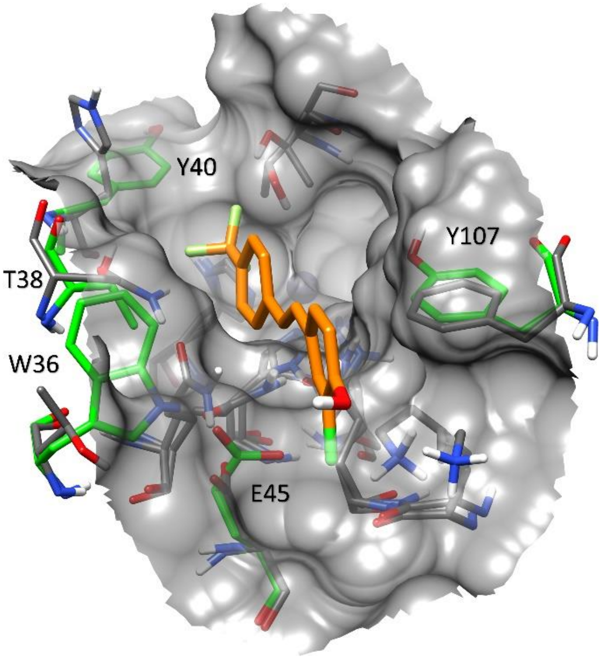

Figure S6. Minimized average structure of hCA VII (PBD code: 3MDZ) in complex with compound **4** superimposed to hCA VA homology model. hCA VA non-conserved residues are labelled and shown in green. The other residues of hCA VA and hCA VII are shown in grey. The protein surface is shown in dark grey.

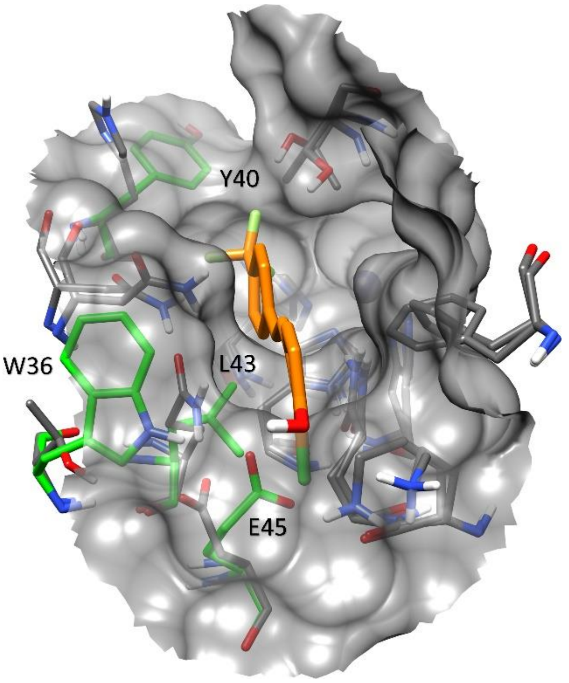

Figure S7. Minimized average structure of hCA VII (PBD code: 3MDZ) in complex with compound **4** superimposed to hCA VB homology model. hCA VB non-conserved residues are labelled and shown in green. The other residues of hCA VB and hCA VII are shown in grey. The protein surface is shown in dark grey.

Table S1. Linear Interaction Energy (aLIE) results for the four analyzed ligand-protein complexes of compound **4**. Electrostatic (EELE) and van der Waals (EVDW) contributes are also reported. Data are expressed as kcal/mol.

Complex	EELE	EVDW	aLIE
hCA II	-0.8	-24.3	-25.1
hCA IX	-2.3	-25.0	-27.3
hCA VII	-7.5	-25.5	-33.0
hCA XII	-5.7	-24.4	-30.1
